# Evidence to support integrating feedback best practice for computer‐based assessment in pharmacology education

**DOI:** 10.1002/bcp.70302

**Published:** 2025-09-29

**Authors:** Claire Y. Hepburn

**Affiliations:** ^1^ School of Medicine University of St Andrews UK; ^2^ Aston University Birmingham UK

**Keywords:** clinical education, computer‐based assessment, feedback, pharmacology education

## Abstract

Feedback is the most powerful driver of learning, but it can afford variable effects depending on the method used. The design of feedback for computer‐based assessment—now increasingly prevalent in higher education—remains relatively underexplored, particularly for pharmacology education. The growing momentum of curricular evolution in pharmacology education presents a timely opportunity to appraise and improve feedback practices in computer‐based assessment within the discipline. While there is extensive literature which outlines the principles of effective feedback, there is not a universal model we can apply with consistently positive outcomes. This review explores how empirical evidence can inform the development of effective feedback for use in computer‐based assessment. Drawing on findings from multiple meta‐analyses and primary research exploring elements of feedback design from across disciplines, this review confirms that elaborate feedback is more effective than corrective feedback alone. However, to support students to develop metacognitive skills for life‐long learning, feedback should be tailored to the characteristics of the learner with dialogic support necessary to optimize impact. It is also clear that a key prognostic indicator of successful feedback for performance and satisfaction is clear links to goals and standards. This review argues for feedback for computer‐based assessment to go beyond that which is efficient. Instead, it should be constructively aligned with clear goals, and be learner‐centred and dialogic to ensure we support the development of reflective and capable graduates of pharmacology education.

## 1 INTRODUCTION

Feedback is widely recognized to be the single biggest driver of learning, providing not only information about performance but also supporting development of metacognitive skills which drive future learning. Variable effects are observed for different approaches to feedback as described in meta‐analyses.^1^, ^2^, ^3^ Over the past decade, there has been an increase in the use of computer‐based assessment with evidence in support of their integration in our curricula.^4^, ^5^, ^6^, ^7^

With the increase in use of computer‐based assessment and the recent momentum pushing evolution of pharmacology education, we must naturally turn our attention to how we can apply the corpus of research on effective feedback to develop feedback for computer‐based assessments in the discipline. This review outlines the principles of effective feedback including timing of feedback, use of elaborate and motivational feedback and how this might be applied in practice to support development of students in pharmacology education.

Feedback is widely recognized to be a critical motivator for effective learning. Hattie and Temperley describe feedback as a consequence of performance^8^ and effective feedback functions not only to inform students about their approach to learning but helps to improve their performance during summative assessment tasks. Despite the extensive literature in the development of effective feedback, a universal ‘magic bullet’ approach to its design is yet to emerge with meta‐analyses reporting variable effects to different approaches.^2^, ^9^

In parallel, assessment in higher education increasingly relies on use of computer‐based assessment, in part a product of increasing cohort sizes and the scalability of more traditional assessment formats.^10^ There is an extensive corpus of evidence regarding the utility of computer‐marked assessments of various formats, including for competency‐based programmes, like medicine,^4^, ^5^, ^6^, ^7^ in addition to those describing requisites for effective feedback, but there is less information regarding feedback for computer‐marked assessment, particularly in pharmacology education despite performance on iterative formative computer‐based assessment improving with provision of feedback.^11^ Naturally, therefore, we must ask, what evidence exists for design of feedback for computer‐based assessment to support growth of capable and competent pharmacology graduates?

The aim of this review is to outline the principles of effective feedback and propose how this might inform feedback design for computer‐based assessment in pharmacology education.

## 2 KEY FEATURES OF GOOD FEEDBACK

For the purposes of this review, computer‐based assessment describes any content‐rich assessment format delivered digitally, including, but not limited to, the following question formats; multiple‐choice, single best answer, extended matching, very short answer, hot spot and/or key feature. Feedback is assumed to occur formatively to facilitate student development towards a summative assessment.

For the purposes of this review, computer‐based assessment feedback (also referred to as computer‐based instructional feedback) encompasses feedback covering simple error flagging (a guide for students about whether their answer was correct or incorrect in absence of any additional information) or, correctness indicators which go beyond simply indications of where errors exist to also include the correct response and finally, feedback which might include either of those plus scaffolding to additional reading material or hints. This type of feedback has traditionally been automatically deployed by the assessment platform with varying degrees of academic input needed for its curation. It is acknowledged that increasingly, this work could evolve to better meet the developmental needs of the student with the use of generative AI but this review focuses on primary peer‐reviewed literature published prior to the wholescale use of AI‐facilitated feedback approaches.

Feedback, in the broadest form, is widely acknowledged to be a powerful influence on learning with an overall effect size of 0.73 (*d* = 0.73; SE = 0.061), which would be described by Hattie as advancing learning in the experimental cohort, exposed to feedback, by almost 9 months and represents 2050 effects from a sample of 1287 studies run from 1980–2006 across a breadth of educational settings.^1^, ^2^, ^12^ Computer‐assisted instructional feedback has a comparatively smaller effect size of 0.52, representing 129 effects, reported in Hattie and Temperley's influential work on the power of feedback.^8^ Again, Hattie might describe computer‐assisted feedback as advancing learning by over 6 months. Despite the smaller effect size, systematic reviews conclude that most students are positive about computer‐assisted instructional feedback^13^ and some evidence suggests interventions help to facilitate task‐focused feedback recipience.^3^ Moreover, student perceptions of computer‐based assessment feedback are positive.^14^, ^15^

Feedback for computer‐based assessment, will often default to corrective feedback, i.e. simple indicators of correctness, which support recall^16^ but is less likely to support the development of self‐regulation via external feedback since this requires reflection.^17^ The constructivist interpretation of feedback, as posited by Nicol and Macfarlane‐Dick, emphasizes that effective feedback should support students to reflect on their current performance relative to established standards. By supporting this reflection, feedback can help students identify the gap between their current performance and their desired level of achievement, from where they can engage self‐regulation and metacognitive processes to plan to close the gap.^17^ Therefore, a requisite of good feedback requires a format conducive to this, as a minimum. Indeed, when students report their experience of assessment, even in those who make good use of formative assessment opportunities (66.5–81.6% of *n* = 496 year 2 students used formative quizzes in a core module captured using virtual learning environment use log data), the same students describe difficulty in understanding goals and standards reported using the Assessment Experience Questionnaire, as described in a cross‐sectional study of students in years 1–3 of a Biomedicine programme in Australia.^18^, ^19^ This suggests that even engaged students might struggle to understand what they are aiming for. These clear goals, should of course be facilitated by appropriate constructive alignment between assessments and taught learning outcomes. Regarding feedback on computer‐based assessment, elaborate feedback, which includes some form of explanation, for example hints or extra reading material, produces larger effect sizes (*g* = 0.49; 90% confidence interval [CI] [0.36–0.62]) as compared with corrective feedback of any form (knowledge of results (*g* = 0.05; 90% CI [−0.28–0.39] and knowledge of correct response (*g* = 0.33; 90% CI [0.02–0.63]) as reported in a meta‐analysis focused on quantitative improvement in achievement for three different styles of item‐based feedback in computer‐based assessment in a sample of studies primarily from college, university or adult education.^20^ A non‐crossover study exploring student engagement with elaborate feedback delivered by an assessment platform suggested students—in cohorts from commerce, law and health programmes—focus their attention to formative feedback where it pertains to elements where they performed poorly.^21^ This reinforces the need for feedback to have a clear goal orientation if it is to support improvements in future summative performance. This trend was also observed in first‐year medical students in an Australia MBBS programme who received non‐elaborate feedback focused on domain‐level performance and who indicated a preference to revisit the incorrect questions so they could focus revision.^22^ Taken together, it would suggest that feedback with clear goals and standards and information relevant to constructs will support the greatest learning gain and be well received by students.

Effective feedback must also endeavour to motivate and positively ideate the student because feedback intervention and its effects on performance are in part a product of personality.^3^, ^8^, ^17^, ^23^ To achieve this, feedback can influence a student's learning orientation. Shute synthesized evidence which showed that feedback can positively influence learning orientation when it scaffolds development of self‐regulation, for example by coaching metacognitive strategies.^24^, ^25^ Some scaffolding approaches mirror the emerging characteristics of large language model outcomes, which can provide process‐orientated feedback. However, the effect size of motivational feedback interventions, related to intrinsic motivation and self‐efficacy (*d* = 0.33; 95% CI [0.23–0.42]) and behavioural feedback interventions, related to behaviour in the educational setting (*d* = 0.48; 95% CI [−0.09 ‐ ‐1.06]) in any recognized educational setting (kindergarten to university) described by Wisniewski's meta‐analysis of 435 studies representing 994 effects sizes, are small or medium, perhaps a product of limited focus of the meta‐analysis, and might caution porting of feedback strategies aimed at these outcomes.^9^ So, rather than attempting to apply a generic approach, it might be more appropriate to understand the characteristics of the feedback recipient, specifically, understanding a student's intrinsic feedback orientation. Feedback orientation is a multidimensional quasi‐trait, stable for approximately 6 months, which includes domains like self‐efficacy, social awareness, accountability and utility and might be useful in tailoring student feedback for computer‐based assessment.^26^, ^27^, ^28^ Because feedback orientation or feedback methods can influence how feedback is interpreted by a recipient, tailoring feedback to align with the needs of the individual may enhance how effective it is for computer‐based assessments.^13^, ^29^, ^30^ In practice, this suggests that we should consider designing feedback that is sensitive to a student's characteristics, potentially using appropriate diagnostic tools or analytics embedded in virtual learning environments, freely available in most institutions who teach pharmacology, to assess feedback orientation and adapt feedback to the individual or sub‐groups of individuals.

The timing of feedback—immediate or delayed—has been the subject of considerable review, with mixed findings across studies.^10^, ^31^, ^32^, ^33^ Meta‐analyses indicate that immediate feedback is best suited for lower order learning outcomes, like procedural or motor skills,^25^ rather than the higher order skills we might expect in competency‐based programmes in pharmacology education. Nevertheless, immediate feedback can be the preference for students for complex tasks like programming, which therefore does not preclude its use in pharmacology education where students complete tasks like calculating elimination rate constants in different compartment models.^34^ Interestingly, in a small study (*n* = 41) of retention of constructs from immediate (feedback after each question) *vs*. delayed (feedback after test item group) elaborate feedback on near and far transfer single best answer questions focused on diagnostic reasoning found no effect of feedback timing in a cohort of second‐year graduate entry medical students.^11^ A more comprehensive study by van der Kleij et al. noted that there was no difference in summative assessment performance for either students who had immediate corrective and elaborate feedback, delayed corrective and elaborate feedback or only a delayed grade with no corrective feedback; rather, the primary predictor of performance is student characteristics in a cohort of first‐year commercial economics students (*n* = 152) in the Netherlands.^32^ Despite the findings, students report elaborate feedback valuable, suggesting that construct type and quality of feedback matter more than timing.^10^ Finally, for those concerned about policy‐mandated feedback timing and prediction of student satisfaction with assessment and feedback on their programme of study, there is only a weak negative correlation in a secondary analysis of UK university policies on feedback turnaround and student satisfaction of assessment and feedback reported via the National Student Survey (NSS).^35^ This suggests that what should drive timing of feedback release is acceptability to academics, the assessment type and cohort, rather than policy. In developing computer‐based assessment feedback, the focus should be on providing content‐rich, constructively aligned feedback delivered at a time acceptable to students and instructors.

A limitation of the discussion thus far is that for any feedback to be effective and influence a student's future performance, the student must actively engage with their feedback.^8^, ^13^, ^36^ The locus on control must lie with the student, as thoughtfully explored by Boud and Molloy.^37^ Student engagement with feedback is multi‐dimensional and argued to be underpinned by four distinct constructs: behavioural, cognitive, social and emotional, and there is zeitgeist to use tools which explore these constructs to tailor feedback to ensure it is most effective for students.^13^, ^38^ For feedback to genuinely facilitate self‐regulation, students must not only receive feedback but need to proactively seek and engage with it in dialogue with instructors.^17^, ^37^ However, in the context of computer‐based assessments—often chosen in part for their efficiency in large cohorts—opportunities for this dialogue are limited.^33^ Van der Kleij identified that computer‐based assessment feedback which combines metacognitive information together with performance and correctness information produces greater effect sizes as compared with both no feedback or corrective feedback of any form, as described earlier.^20^ This might be in part a product of how accessible the feedback is. Gordijn and Nijhof noted that students with weaker literacy benefitted less from complex feedback than those with stronger literacy skills, suggesting complementary methods to support engagement with feedback for these students might be beneficial.^39^ Therefore, development of good feedback on computer‐based assessment requires a holistic curriculum‐level solution to attempt to address the 2 sigma problem by supporting the feedback dialogue element of one‐to‐one tutoring whilst still being scalable and sustainable.^37^, ^40^ In practice, this would involve systematic introduction of feedback review sessions with academics during instruction or in personal tutor sessions.^41^ By intentionally building structured time to reflect and engage in dialogue on feedback with the support of instructors, we can avoid the passive receipt of feedback that often plagues computer‐based assessment feedback.

Finally, an oft overlooked element of computer‐based feedback design in the literature is how conceptual misunderstandings, highlighted by formative and summative assessments, influence strategies for interventional teaching or annual teaching review, or changes in approaches to feedback.^17^ There is no doubt that instructors are practising these metacognitive and reflective processes in the course of their work but the formalization across a curriculum or even review of new computer‐based assessment feedback may be limited if the number of peer‐reviewed publications are indicative of practice.^20^, ^42^ Lessons from the literature on feedback for more traditional assessments offers several examples of the approaches to support these reviews.^11^, ^16^, ^33^

## 3 WHAT THIS MEANS FOR COMPUTER‐BASED ASSESSMENT FEEDBACK IN PHARMACOLOGY EDUCATION

Pharmacology education for those who wish to enter clinical practice is competency‐based and, in addition to satisfying the requisite knowledge‐related components of a course, feedback on assessments can help students to develop the necessary metacognitive skills to become effective life‐long learners.^43^ Since August 2016, it became a requisite for foundation year 1 (F1) trainee doctors (a type of resident doctor) enrolled in the Foundation Programme in the United Kingdom (UK) to pass the Prescribing Safety Assessment (PSA), an assessment designed to allow demonstration of competence in the safe and effective use of medicines in clinical practice.^44^ The development of the PSA was prompted by several reports noting concerns in prescribing practice and the preparedness of foundation years trainee doctors for clinical practice. For example, an empirical study of prescribing errors made by F1 trainee doctors reported a prescribing error rate of 8.4%.^43^ A small systematic review on perceptions of preparedness for clinical practice highlighted prescribing and taking responsibility for their learning as areas of concern for trainee doctors. However, factors like early exposure to complex material through a vertically integrated curriculum and recognition of personal limitations positively influenced perceptions of preparedness, suggesting a possible role for feedback in facilitating improvements in perceptions of preparedness in these cohorts.^45^ Finally, a rapid review on preparedness of all graduates from UK medical schools from literature published between 2009 and 2014, similarly found that trainee doctors are not prepared for safe prescribing and in a follow‐up multi‐centre qualitative study by the same authors, they found stakeholders reported postgraduate year 1 doctors (synonymous with F1 trainee doctors) lacked basic understanding of pharmacology.^46^, ^47^ The issue is also not limited to the UK; a systematic review of the international literature also identified inadequate competence in prescribing in final year medical students.^48^ It is clear that perceptions of preparedness to prescribe and safe prescribing practice were a problem and while an independent review of the PSA did not have robust data to measure changes in prescribing errors since roll‐out, the authors report normalized patient safety incidents and referrals for prescribing errors, reported via the National Reporting and Learning System and General Medical Council (GMC), respectively. Despite the increase in educational interventions to teach pharmacology, which sat alongside the roll‐out of the PSA, approaches to explicitly address mechanisms for providing feedback to students were often absent.^49^, ^50^ Importantly, pharmacology education extends far beyond medical curricula, playing a critical role in the professional development of students in biomedical sciences, pharmacy and related disciplines. The challenges identified in medical education may be the canary in the coal mine and point to a need to appraise and review pharmacology education more widely. Given the speed and volume of adoption of computer‐based assessment and the emerging momentum for curricular reform in pharmacology education—first with agreement of curricular content in clinical pharmacology education in Europe in 2018, which included recommendations for use of computer‐marked assessment for early years and more recently with the development of core concepts in pharmacology supported by the International Union of Basic and Clinical Pharmacology (IUPHAR), which aim to develop future students who can manage complex and novel pharmacology problems more readily—this is a timely opportunity to integrate evidence‐based feedback approaches into computer‐based assessments that support metacognitive skills development and formation of competent, successful graduates of pharmacology.^51^, ^52^, ^53^, ^54^ The evaluation of key core concepts in pharmacology, amongst undergraduate students internationally pointed to gaps in understanding and may further support the need to appraise and improve feedback practices given how effective feedback can be in learning.^55^, ^56^ So, how can we apply the corpus of evidence which describes effective feedback to the development of feedback for computer‐based assessment feedback in pharmacology education?

**FIGURE 1:**
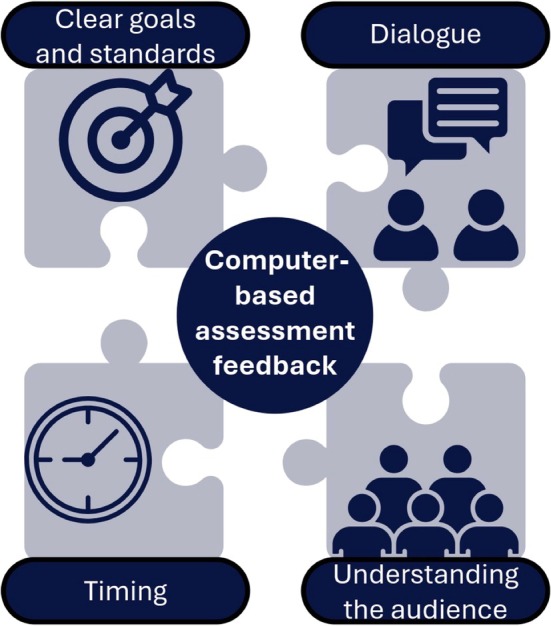
The key components of effective feedback for computer‐based assessment in pharmacology education includes clear goals and standards, opportunities for dialogue with an appropriate academic in a cohort or individual setting, timing which recognizes the needs of the local context, and feedback content that recognizes the needs of the intended audience.

The key elements of effective feedback include clear goals and standards, appreciating the needs of the intended audience, appropriate timing and supporting dialogue (Figure 1).

### 3.1 Clear goals and standards

Feedback for computer‐based assessment must constructively align taught learning outcomes with assessed standards, ideally on a delivery platform that supports learning outcomes tagging. Simple indications of correctness, often provided as default feedback on assessment platforms, is inappropriate; elaborate feedback is optimal.^17^, ^18^, ^19^, ^20^

### 3.2 Understand the audience

Feedback should positively ideate students to encourage self‐regulation. To achieve this, it is important to know your audience and pitch the feedback to achieve this. Moreover, ensuring that impact of this approach is carefully analyzed post‐hoc. The emergence of large language models may now make a more individual approach to feedback achievable.^8^, ^17^, ^23^

### 3.3 Timing of feedback

This should be informed by the local context and the learning outcomes being assessed. Student perceptions of immediate feedback are positive but there is variable evidence that this translates to summative assessment improvement.^10^, ^31^, ^32^, ^33^

### 3.4 Support dialogic feedback

Careful review of the curriculum to support integration of efficient and sustainable dialogic feedback sessions to facilitate development of metacognition.^33^, ^37^, ^41^

## 4 PERSPECTIVES

As we continue to adopt computer‐based assessment in pharmacology education and as the curriculum rapidly evolves, there is a pressing need to ensure that the feedback from this assessment format does more than simply indicate correctness; it must meaningfully support learning.

This review highlights the evidence that indicates that feedback from computer‐based assessment should be elaborate, goal‐orientated and sensitive to the individual needs of our students. There are mixed results regarding the benefits of immediate or delayed feedback and so the timing should be informed by the local context. Embedding opportunities for dialogue between instructors and students following feedback, either within instructional sessions or via a personal tutor, may help bridge the gap between scalable delivery and personalized learning. Finally, feedback should not be seen as a terminal activity but as an active tool for informing teaching practice and holistic curriculum design. In practice, we must ensure feedback for computer‐marked assessment is not simply efficient but educationally effective and, in doing so, support the development of capable, reflective pharmacology graduates.

## CONFLICT OF INTEREST STATEMENT

There are no competing interests to declare.

## Data Availability

None.
